# Boosting the Economic Recovery or Closing a Green Deal in Europe? Or Both?

**DOI:** 10.1007/s10272-020-0930-0

**Published:** 2020-12-01

**Authors:** Natacha Valla

**Affiliations:** Sciences Po School of Management and Innovation, 9, Rue de la Chaise, 75007 Paris, France

## Abstract

The recovery measures announced by Europe in parallel with the Green Deal are the perfect opportunity to step up the effort to measure the impact of public expenditure, both in the short and in the long term. To that aim, it is essential to quickly stabilise benchmarks to measure the financial and non-financial impact of investments in the European recovery plan.

In 2020, the EU gave birth to the largest stimulus package it ever funded. Between the long-term budget of the EU and the temporary instrument to boost recovery (Next Generation EU), a global envelope of €1.8 trillion is being mobilised to help rebuild post-COVID-19 Europe. The agenda is simple: "a greener, more digital and more resilient Europe".[1]

## The specific economic profile of the COVID-19 crisis

From an economic perspective, the crisis that unfolded after the outburst of the COVID-19 pandemic has been particular in many ways. Its specificities dictate the way in which any recovery plan should be designed and implemented.

A key specificity of the 2020 crisis is the intensity with which the service sector has been affected. While the manufacturing and construction sectors have suffered the most from recent cyclical downturns, this time – particularly the lockdown of spring 2020 – they were more resilient than the service sector. During the first half of the year, the contribution of the service sector to the contraction of GDP was three times greater than that of the manufacturing sector in the euro area.

1 As stated on the European Commission website, https://ec.europa.eu/info/strategy/recovery-plan-europe_en, on 10 November 2020, the European Parliament and the EU countries meeting in the Council reached agreement on the next long-term EU budget and Next Generation EU. This agreement will strengthen, with an envelope of €15 billion, specific programmes within the framework of the long-term budget for the period 2021-2027. But follow-up discussions by member states have evidenced political difficulties that could arise through veto positions.

**Natacha Valla**, Sciences Po School of Management and Innovation, Paris, France.

As a result, the heaviest burden today is borne by the lowest paying jobs in the service sector. This is true both in the United States and in Europe. In the United States, minorities and women are over-represented in this employment sector and will therefore pay the heaviest price. In Europe, too, job losses have disporportionately affected different socio-economic groups. Over the first half of 2020, the working population shrank by 7% for low qualifications, while it increased by more than 3% for the highest qualifications, as highlighted by the ECB.[2]

In general, economists consider that over a long period, technological progress is associated with an increase in total factor productivity. However, in the short term, it is difficult to confirm this observation. The jury is still out. And once the crisis has passed, we will have to find our way back closer to full employment.

A second specificity of this recession is the nature of the shock that hit the European economy. Many of the technological developments that the crisis dramatically accelerated were in the making. But our deliberate shutdown of the economy at a global scale produced a very unusual recession. Some may recall that: in the first few weeks of the pandemic, economists struggled to figure out whether it should be characterised as a supply shock or a demand shock. More than a rhetorical debate for specialists, this was a fundamental point because each calls for a very different economic policy reaction. For my part, with hindsight, I very much like the idea put forward by Guerrieri et al. (2020), that the shocks that followed the pandemic are 'Keynesian supply shocks'. The lockdowns are most certainly initially supply shocks due to the incompleteness of the markets. But then, recent (and future) layoffs and bankruptcies will amplify the consequences of initial shocks according to a Keynesian logic of declining demand.

2 See the keynote speech by Christine Lagarde, President of the ECB, at the ECB Forum on Central Banking, 11 November 2020 (European Central Bank, 2020). This section is based on an interview I gave to Atlantico on 16 November 2020 (see Valla, 2020).

## Common fiscal stimulus is indeed the best policy response

International institutions and central banks speak in unison: we must urgently pursue a coordinated policy mix and the role of fiscal policy must be maximised. Central bank monetary easing has been expanded everywhere. However, it does not mean that public money should be spent carelessly, on the contrary. The means that member states earmarked under the Next Generation EU flag should be allocated carefully. It tends to take longer to emerge from recessions that most intensely impact the service sector: We will not go to the hairdresser twice as much once the lockdown is over! In addition, since services are more employment-intensive than manufacturing, the eventual impact of the current crisis on employment should be expected to be particularly severe. Hence the need to target public support to the labour market in the short term.

As the ECB pointed out, five million Europeans lost their jobs in the euro area in the first half of 2020. Half of these jobs were in sectors that represented only 20% of GDP including retail, trade or transport. Cyclical measures to ease the contraction in demand and make it possible to hold out during difficult times are certainly needed. But they can also be accompanied without procrastination by a more structural approach to help certain sectors with reconversion.

In the short term, we obviously have to show solidarity and protect the most vulnerable populations – and not just workers. And day after day, we are discovering that economic vulnerabilities are compounded by serious social and psychological suffering. Public spending through social transfers is best suited to this situation. The pandemic has forced us to temporarily suspend an entire part of economic activity, which has interrupted the circularity of payment flows. It is therefore a targeted intervention that will be most effective. As Woodford (2020) aptly states, fiscal policy can, under similar circumstances, achieve the best results without monetary policy intervention necessarily being helpful.

But in the design of public intervention, one must also have the courage to look to the future. The most effective long-term measures are those that have been able to support the wide range of changes brought about, or hastened by the pandemic, such as the acceleration of the digitisation of human spheres of activity, and renewed attention to climate issues. Education, training and other measures supporting the employment of tomorrow are essential.

Stating Woodford's position more moderately, on the monetary policy side, it is important to note that central banks in the euro area and in the US are in line with this analysis and loyal to the post. Their role remains to support inflation dynamics by supporting demand, thereby preventing second-round deflationary effects from emerging on prices and wages. It is up to them to maintain favourable financing conditions, which they do.

## Bridging Next Generation EU and the Green Deal

To satisfy the generational aspiration for more sustainable and inclusive prosperity, Europe today has the strongest potential for renewal. As Baptiste Perrissin-Fabert and I argued recently,[3] the stars are aligned. First, with the recovery plan, Europe has acquired a new (revolutionary!) capacity for issuing common public debt. Moreover, the massive purchase of public debt by the Eurosystem has propelled central banks to the center of the handling the COVID-19 crisis with unprecedented power. Finally, with all due respect to the 'frugal' countries, the Maastricht budgetary rules have been suspended indefinitely, which saves time in the short term. We should be delighted that European leaders have succeeded in going beyond the limits of yesterday: This is the optimal time to build our collective resilience in the face of the climatic, health and social crises that threaten our societies.

To start with, Europe should not be lured by overly accessible liquidity. While the current environment makes new debt possible and seemingly harmless, that in itself is not good news. COVID-19 debt will be added to mountains of existing debt for which the conditions of sustainability will have to be secured over time. The debate amongst economists on the remedies to manage this debt is lively: cancellation, monetisation, rollover in perpetuity, exceptional taxes, forced investments, ten-year austerity.

## Good debt, bad debt and impact evaluation

It is too early to settle this debate, but Europeans should be aware that a decision will need to be made when the time comes. But let us set aside these theoretical options for the moment to focus on the more operational challenge of using this new European common debt. We believe, like former ECB President Mario Draghi, that there is "good" and "bad" debt. How do we avoid the déjà vu of wasted money, hastily recycled project funding, or wasted funds on infrastructure of questionable utility?

Times have changed. A new generation is rising, making a tangible impact in terms of environmental and social

3 OpEd in Les Echos, 24 November 2020, on which the sequel is based.

sustainability. This generation claims "good" debt. More and more companies and investors are sincerely committed to putting impact at the heart of their business model. Europe must seize on these fundamental movements to make them a project of innovation for the best, of solidarity between its member countries, of economic sovereignty and of environmental power.

The recovery measures announced by Europe in parallel with the Green Deal are the perfect opportunity to step up the effort to measure the impact of public expenditure, both in the short and in the long term. To that aim, it is essential to quickly stabilise benchmarks to measure the financial and non-financial impact of investments in the European recovery plan. When choosing between the financing of road infrastructure, technological innovation in telecoms or the renovation of schools, it is no longer possible to put on blinders and focus solely on economic and financial profitability. Impact evaluation must take into account everything that today "does not count for accountants", in particular the extra-financial impacts that will, for example, make it possible to increase human capital (health, education), or even natural capital (avoided $CO_2$ emissions, services provided by biodiversity).
